# Impact of hatchery microclimate, breeder age and storage time on egg water loss in a tropical climate

**DOI:** 10.1007/s00484-026-03332-z

**Published:** 2026-10-05

**Authors:** Aérica Cirqueira Nazareno, Robson Mateus Freitas Silveira, Afrânio Márcio Corrêa Vieira, Iran José Oliveira da Silva

**Affiliations:** 1 Department of Biosystems Engineering, Luiz de Queiroz College of Agriculture (ESALQ), University of São Paulo (USP), Piracicaba, São Paulo 13418-900 Brazil; 2 Department of Statistics, Federal University of São Carlos (UFSCAR), São Carlos, São Paulo 13565-905 Brazil

**Keywords:** Ambience, Poultry farming, Thermal comfort, Incubation

## Abstract

This study aimed to evaluate the effects of incubator microclimate, broiler breeder age, and fertile egg storage duration on egg water loss in a commercial hatchery within a tropical climate. Data were collected from the environments (air-conditioned egg room and incubator) in order to perform microclimatic assessments, in which temperature (T; °C), relative humidity (RH; %) and specific enthalpy (h; kJ.kg of dry air^− 1^) were recorded at 10-minute intervals. Twelve Logen microprocessors were used in the egg room and one microprocessor was assigned for every three treatments in the incubators. Water loss in fertile eggs was verified using eggs from three different ages of Cobb Fast breeders (young = 29–30 weeks, intermediate = 42–43 weeks, and old = 52–53 weeks), from the same flock and analyzed over time. Twelve storage time ranges were also analyzed (87–103, 174–184, 196–208, 244–255, 63–77, 150–160, 172–184, 220–231, 40–57, 128–137, 150–161 and 198–208 h). In each treatment (combining breeder age and storage time), 35 individual eggs were weighed, totaling 1,440 eggs. Weighing was conducted upon entry and exit from both the egg storage room and the incubator using a semi-analytical balance. The experimental design used was hierarchical, in which the storage times are nested within the ages of the broiler breeders, with 35 replicates. T, RH, and h in the egg room and incubator showed higher averages during summer (*p* < 0.0001), and remained outside the recommended thermal and enthalpy ranges across all seasons under tropical conditions. The highest water loss of fertile eggs in the egg room and incubator was observed in eggs from older breeders (*p* < 0.0001) and under longer storage periods (*p* < 0.0001), particularly at 244–255, 220–231, and 198–208 h. Under tropical conditions, the T and h of the egg room and incubator are not within the ideal ranges in the studied seasons. Eggs from old breeders and those with longer storage times were those which presented the greatest water loss values. These findings highlight the need for improved environmental control in hatchery facilities under tropical conditions, particularly through tighter regulation of temperature and humidity and by minimizing egg storage duration, especially for eggs from older breeders, in order to reduce excessive water loss and optimize incubation performance.

## Introduction

Hatcheries have been seeking to improve the quality of broiler chicks by adopting professionalized management and updating available technologies, especially regarding air conditioning and incubation systems. However, most hatcheries ignore the fact that the egg room requires greater attention in terms of air conditioning systems (Nazareno et al. 2014). After all, what is recorded by the equipment does not always correspond to the ideal air temperature and relative humidity.

In order to ensure that embryonic development is halted and embryo integrity is guaranteed, fertile eggs must be stored in an air-conditioned room for a period of time, which may vary depending on the hatchery’s demand, before incubation. Thus, storing fertile eggs for a short period can improve hatchability; however, direct incubation of fresh eggs results in lower hatchability (Nasri et al. 2020; Dai et al. 2025).

Several studies have shown that prolonged storage of fertile eggs is negatively associated with hatchability (Fasenko 2007; Pokhrel et al. 2018; Melo et al. 2021). Enhancing hatchability reduces incubation costs, improves economic gains, and maximizes the genetic potential of broiler breeders (Dai et al. 2025). The ideal microclimatic conditions for egg rooms should be within the range of 10–20 °C temperature and 55–75% relative humidity (Elibol and Brake 2008a; Pokhrel et al. 2018). However, the ideal temperature for egg incubators is 37.5–37.8 °C, relative humidity of 50–65% (Elibol and Brake, 2008a; Tanure et al., 2009; Abioja et al. 2021, Özlü et al. 2021).

The microclimate of incubators is one of the main factors for embryonic development and metabolism, since during incubation the embryos of birds are poikilothermic (Souza et al. 2025). Understanding these microclimatic conditions helps enhance hatchability and improve broiler chick quality. Water loss from fertile eggs, hatchability and broiler chick quality decrease when the egg storage period exceeds three days, regardless of the temperature (Tona et al. 2001; Özlü et al. 2021). During egg storage, there are a series of changes in the physicochemical aspects, which can cause a decrease in albumen quality (Tona et al. 2001; Pokhrel et al. 2018; Abioja et al. 2021). Furthermore, it has been observed that the stored egg may have a longer incubation period and delayed embryonic development (Reis et al. 1997; Abioja et al. 2021; Özlü et al. 2021).

Egg weight loss during storage and incubation is a key physiological indicator directly associated with embryonic development and hatchability (Silveira et al. 2026). Adequate water loss is necessary to ensure proper air cell formation and gas exchange; however, both insufficient and excessive water loss can impair embryo viability and reduce hatchability. Previous studies indicate that optimal total egg weight loss during incubation typically ranges around 10–15%, as reported in previous studies, depending on breeder age, egg size, and incubation conditions, with deviations from this range leading to reduced hatchability and poorer chick quality (Tona et al. 2001; Reijrink et al. 2010). Therefore, monitoring and controlling egg water loss is essential for optimizing incubation outcomes, even when hatchability is not directly measured.

The age of the breeder is another factor of great importance for egg incubation, since eggs from old breeders must be incubated more quickly than eggs from young breeders (Brake 1996; Zhao et al. 2024). Because young breeders produce better quality albumen, they can withstand a longer storage period compared to those from old breeders (Brake 1996; Feng et al. 2020; Zhao et al. 2024). Young breeders produce smaller eggs with low incubation yield, poorer quality chicks and lower hatching weight (Corrêa et al. 2011; Feng et al. 2020; Zhao et al. 2024). As the matrix ages, the eggs increase in size and there is a greater proportion of yolk in relation to the albumen, due to the greater capacity of old birds to transfer lipids to the egg yolk (Santos et al., 2007; Zhao et al. 2024). There is also a decrease in the thickness of the eggshell, due to the increase in the number and diameter of pores, which favors gas exchange (Agbehadzi et al. 2024; Zhao et al. 2024). Therefore, eggs from old matrices require higher incubation humidity to hinder their excessive dehydration (Costa et al. 2011). Excessive egg dehydration directly affects chick weight and quality at hatching (Tona et al. 2001, 2003; Pokhrel et al. 2018; Abioja et al. 2021).

Therefore, it is imperative to understand the relationship between the microclimatic conditions present in the hatchery’s egg room and incubators, in order to clarify their subsequent effects depending on the age of the breeders and different storage times, assessing their impact on egg water loss.

The objective of this study was to evaluate how the microclimate of the incubators, broiler breeders’ ages and storage times of the fertile eggs affect egg water loss in a commercial hatchery in a tropical climate.

## Materials and methods

**Fig. 1:**
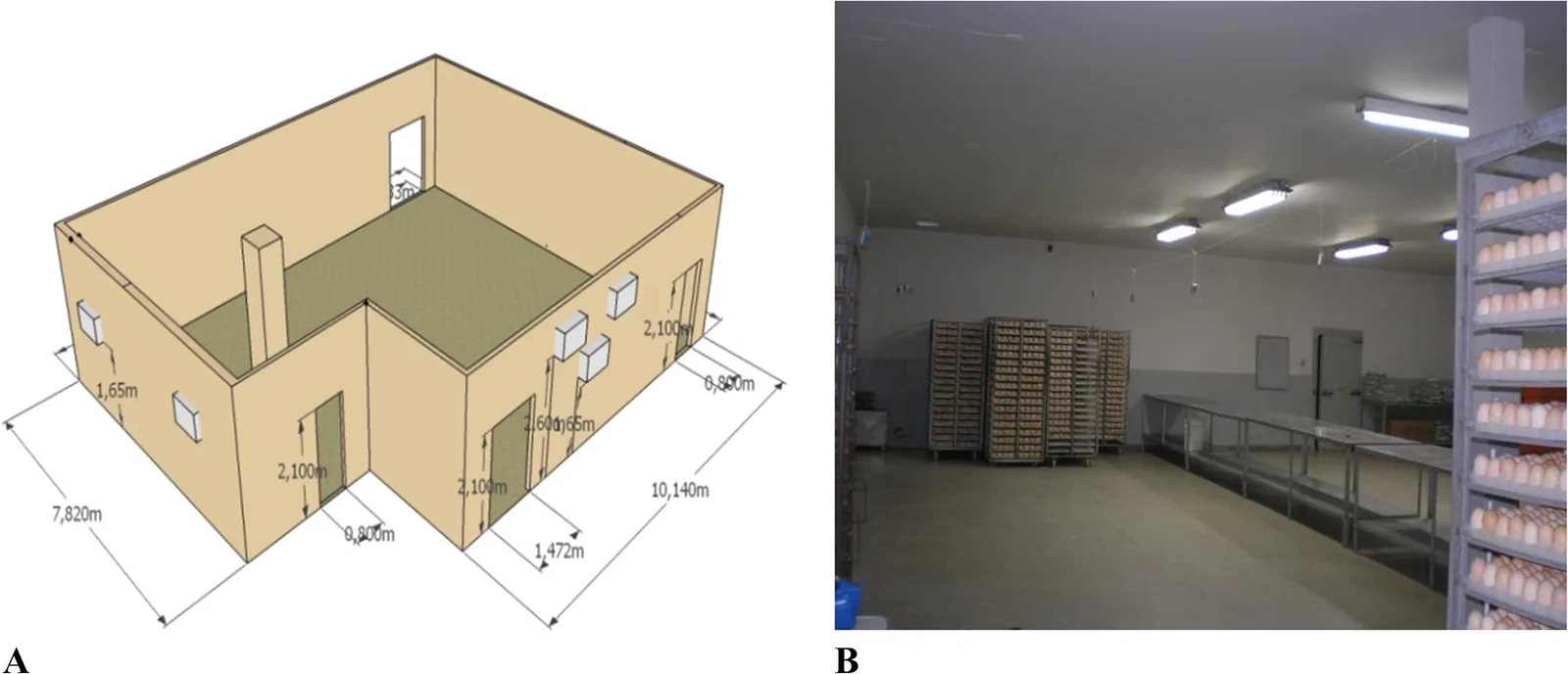
Sketch (**A**) and image (**B**) of the hatchery egg room

This study was conducted at the poultry hatchery facilities of an integrating company, located in the municipality of Mogi Mirim, in the State of São Paulo (22º 25’ 55” S, 46º 57’ 28” W and 590 m sea). The experimental periods occurred in spring, summer and autumn. The climate of the region is characterized as mesothermal Cwa with the temperature of the coldest month under 18 °C, a dry winter and the temperature of the hottest month over 22 °C, according to the Köppen classification (Pereira et al. 2002). The egg room was 13.34 m long by 11 m wide, with a ceiling height of 3 m, totaling an area of 146.74 m^2^, and a volume of 440.22 m^3^ with an east-west orientation, covered with fiber cement tiles, with the presence of a slab; the installation was completely enclosed with masonry, the egg room floor was made of granite, and the space was air-conditioned (Fig. 1).

The air-conditioning system was composed by five air conditioning units, three of which were Springer flex, with dimensions of 0.66 × 0.43 m installed at a height of 2.60 m from the floor, with 80,000 BTUs, and two were Consul, with dimensions of 0.64 × 0.43 m installed at a height of 1.65 m from the floor, with 7,500 BTUs.

The egg cart placement for the different treatments in the hatchery’s egg room was determined based on a preliminary analysis, aiming to position it in areas with microclimatic conditions closest to the ideal ranges (Elibol and Brake 2008a; Pokhrel et al. 2018), with the purpose of promoting microclimate homogeneity in the different treatments. The location of the cart in the egg room was based on thermal mapping of the environment, in which a point sampling network was used, which, through geostatistics, determined the best location. Since there was no spatial dependence, it was decided to locate the cart in a central point of the room.

The microclimatic analyses of the egg room and incubator of the hatchery were performed by recording air temperature (T; °C) and relative humidity (RH; %). The T and RH records were made at 10-minute intervals during the period of permanence in these environments. In order to characterize the heat content in the internal environment of the facility, the psychrometric quantity of specific enthalpy (h; kJ.kg of dry air-1), proposed by Rodrigues et al. (2011), was also used.

The microclimatic variables of the egg room were monitored by 12 Logen microprocessors in the egg room, 6 microprocessors on the egg trays located on the trolley with the appropriate treatments and one microprocessor in the external environment of the facility, at a height of 1.8 m from the floor. Six microprocessors were randomly distributed throughout the six trays to record the microclimate of the evaluated treatments. The incubation trays were placed on the trolley, and these remained in the egg room during the storage periods until they were weighed and transferred to the incubator.

**Fig. 2:**
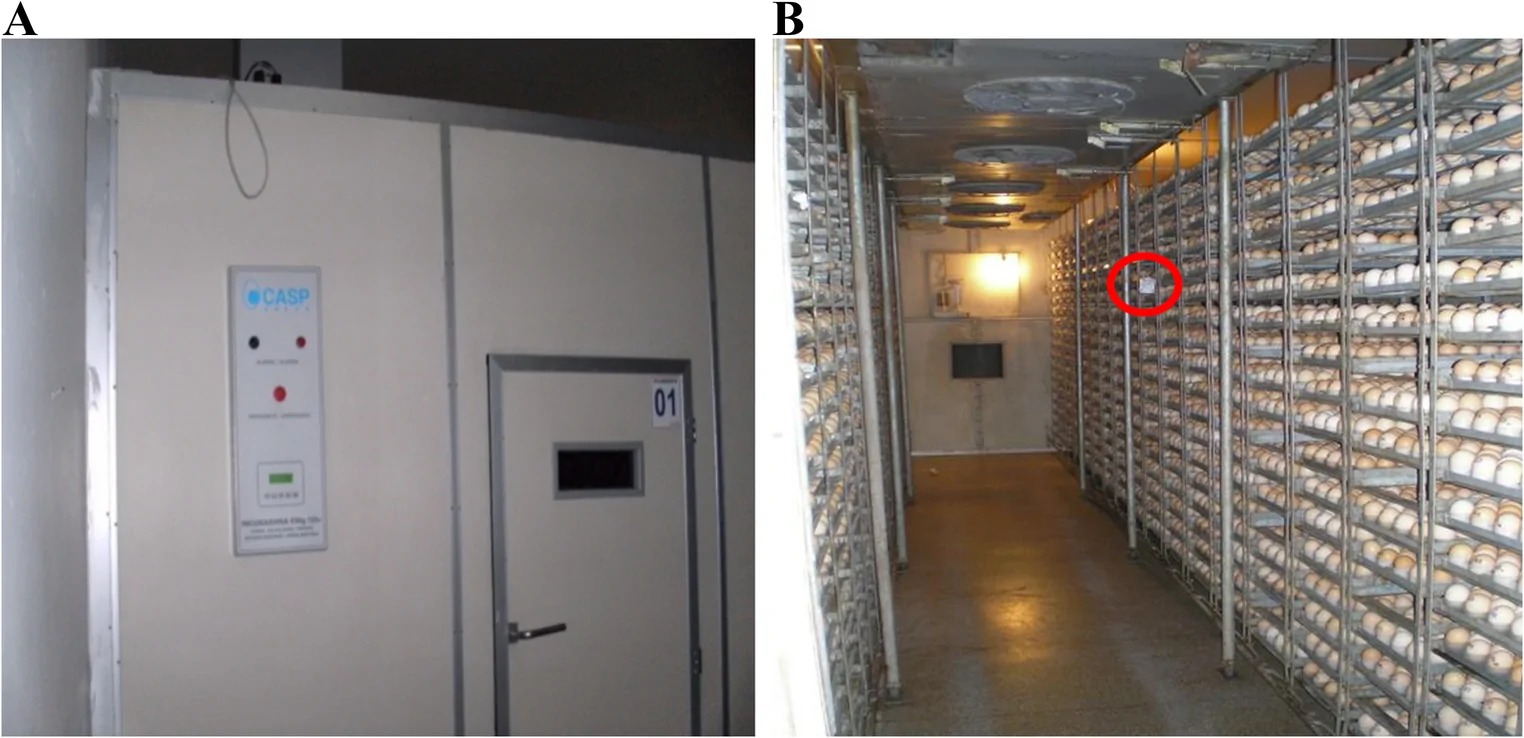
Flowchart of breeder ages and hatchery storage times

This study involved three age groups of Cobb Fast breeders, all from the same flock and analyzed over time, along with their respective storage durations. The age groups analyzed were: young = 29–30 weeks, intermediate = 42–43 weeks, and old = 52–53 weeks It is important to emphasize that each breeder age range was analyzed in three different seasons of the year, namely: young = spring, intermediate = summer and old = autumn. The storage time intervals in the hatchery egg room analyzed were 12 intervals (87–103, 174–184, 196–208, 244–255, 63–77, 150–160, 172–184, 220–231, 40–57, 128–137, 150–161 and 198–208 h). Through these treatments, it was possible to evaluate the influence of the microclimate in the two study environments (egg room and incubators) on water losses from fertile eggs. A flowchart presented in Fig. 2 represents the experimental design.

**Fig. 3:**
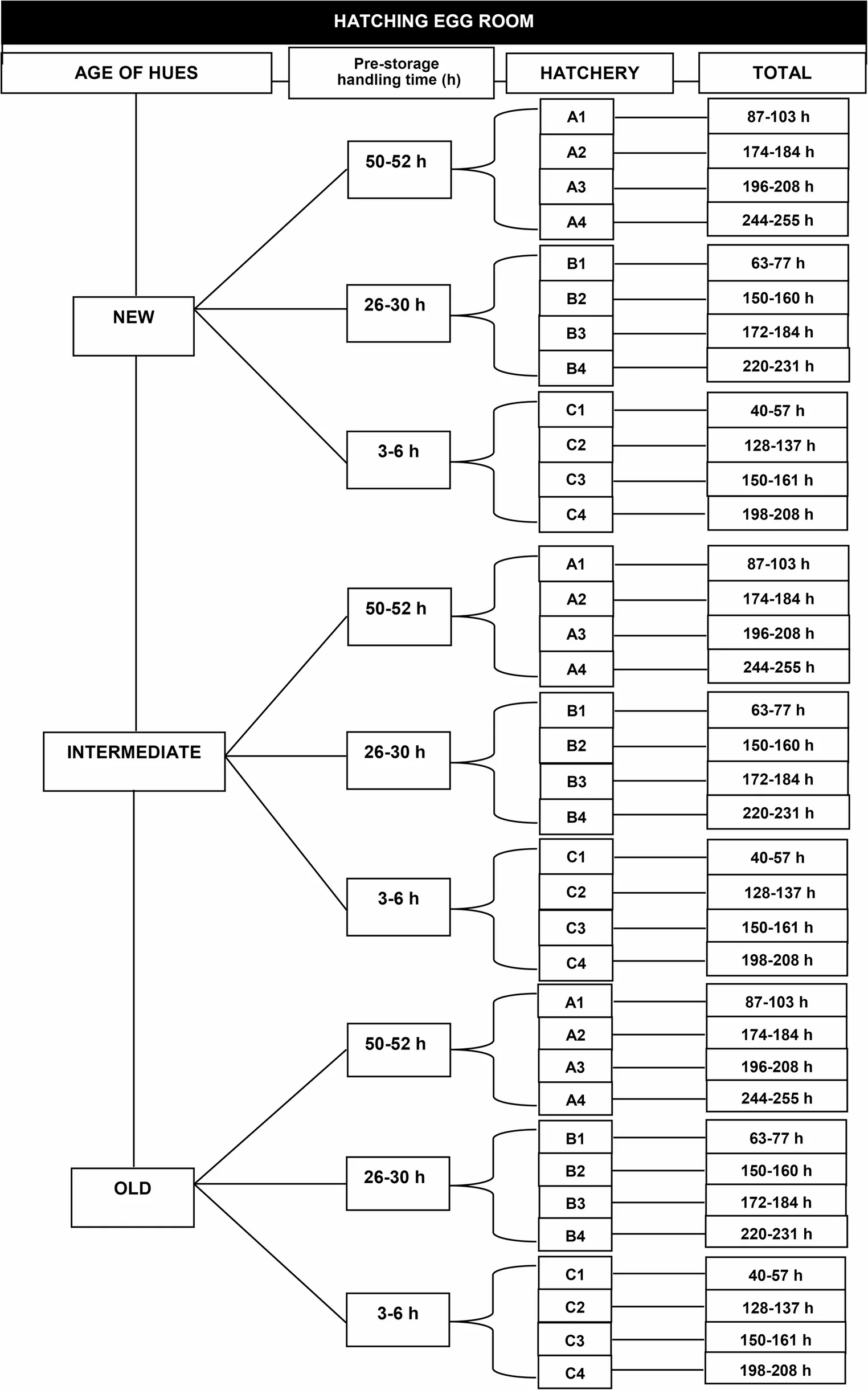
External (**A**) and internal (**B**) view of the incubator and microprocessor allocation (red circle)

A total of 1440 eggs were collected considering the three ages of the breeders, with 480 eggs collected in each phase (young, intermediate and old), which corresponded to six boxes of fertile eggs. The water loss assessment was performed by weighing the eggs (g) individually at the entrance and exit of the two study environments (egg room and incubators) of the hatchery, for each breeder age and 12 storage durations, using a semi-analytical balance with precision of 0.1 g. The incubator was a Casp brand, multi-stage, model CMG 125e, with a total capacity of 124,416 eggs. The incubator had 1,296 drawers, each drawer having a capacity of 96 eggs. The incubator in which the research was carried out had the following dimensions: 3.47 m front, 6.97 m side, 2.68 m height, 24.19 m2 area, and a volume of 64.82 m3. The incubator was air-conditioned with controlled T and RH conditions, as recommended by the authors Elibol; Brake (2008a), Tanure et al. (2009), Abioja et al. (2021), and Özlü et al. (2021) (Fig. 3). The maximum air flow for cooling the incubators was 1,800 m^3^/h.

The incubation trays had a capacity of 96 fertile eggs, resulting in 12 trays per breeder age, corresponding to their respective storage durations. It is important to note that the eggs weighed in the incubator were the same ones identified and weighed in the broiler breeder. There were also three spare trays, which would be used to replace the eggs, if necessary, since the company does not leave empty spaces in the incubation trays. The incubations were always carried out between 3 and 6 a.m. Therefore, three trays were incubated for four days, however, on the fourth day of incubation, the spare trays were also incubated. The entire incubation schedule followed the company’s routine. All eggs were subjected to the same microclimatic conditions (T and RH), lighting, tray turning and gas concentration, during incubation for approximately 18.4 days, totalling 441 h, as recommended by the authors Almeida et al. (2006 a), Elibol and Brake (2008a), Abioja et al. (2021) and Özlü et al. (2021).

In order to record the microclimatic variables, a Logen^®^ brand microprocessor was installed for every three trays of the different treatments. It was located close to the three trays inside the incubator. Therefore, four microprocessors were used inside the incubator and one microprocessor in the incubator room.

After 18.4 days of incubation, the fertile eggs were weighed for each breeder age and their respective storage times. After weighing, the fertile eggs from each treatment were transferred from the incubator into the hatcher simultaneously.

In evaluating the weight of fertile eggs, it was assumed that the microclimates of the hatchery’s egg room and incubator were homogeneous. A hierarchical (nested) design was used, with breeder age as Factor A and storage duration as Factor B nested within breeder age. Each age × storage-time combination included 120 individual eggs. This structure was used because storage durations were specific to each breeder age group, characterizing a nested relationship between factors (Liu et al. 2024).

Regarding the statistical analysis of the microclimate of the hatchery’s egg room, the mixed linear model assuming a first-order autoregressive covariance structure was used, in which the response variables were T, RH and h as a function of the season factor (spring, summer and autumn). It is noteworthy that each season represented a breeder age (spring = young, summer = intermediate and autumn = old). The Tukey-Cramer mean test was used to compare the means.

For statistical analysis of the incubator microclimate, a mixed model with normal distribution was used, in which the response variables were: T, RH and h, and the covariates were: seasons and storage times. The covariance structure is first-order autoregressive. In order to compare the means, the Tukey-Cramer mean test was used.

Egg water loss data were transformed using the logit of water loss. This data transformation is necessary to meet the assumptions the regression model, as it adjusts the loss (which varies from 0 to 100% for the egg room, but for the incubator the values vary from 1 to 100%) to a scale from -∞ to +∞. The logit of egg water loss was calculated using Eq. (1) described below:

1 $$\mathrm{L}\mathrm{O}\mathrm{G}\mathrm{I}\mathrm{T}\_\mathrm{L}\mathrm{O}\mathrm{S}\mathrm{S}=\mathrm{L}\mathrm{o}\mathrm{g}\left(\frac{\mathrm{P}}{1-\mathrm{P}}\right)$$

Where: LOGIT_LOSS = logit of water loss of fertile eggs;

$$P=water\;loss\;of\;egs\;(\%)$$

The inverse of the logistic of water loss of eggs was used to calculate the value of water loss in percentage, described in Eq. (2) below:

2 $$\mathrm{P}=\frac{{\mathrm{e}}^{\mathrm{L}\mathrm{O}\mathrm{G}\mathrm{I}\mathrm{T}\_\mathrm{L}\mathrm{O}\mathrm{S}\mathrm{S}}}{\left(1+{\mathrm{e}}^{\mathrm{L}\mathrm{O}\mathrm{G}\mathrm{I}\mathrm{T}\_\mathrm{L}\mathrm{O}\mathrm{S}\mathrm{S}}\right)}$$

In order to assess the influence of age of the matrices and the storage times on the water loss of fertile eggs in relation to the microclimates, the statistical analysis of the class of mixed linear models was used (Verbeke, 2000; Vieira 2008).

Assuming that the random variables egg weight and microclimate can be modeled by the distribution, the statistical model can be described by the form. (1).

3 $$Y_{i}=X_{i}\beta+Z_{i}b_{i}+\varepsilon_{i}\\b_{i}\sim\;N(0,\;D)\\\varepsilon_{i}\sim\;N(0,\;\sum \nolimits_{i})$$

In which X_i_ and Z_i_ are the design matrices (containing microclimatic factors and/or covariates) for the *p*-dimensional *β* vector containing the fixed effects and the *q*-dimensional vector b_i_ of random effects to model the effect of birds, which has a multivariate normal distribution with mean and covariance matrix D, $\varepsilon_{i}$ is the vector of random errors with multivariate normal distribution with mean and covariance matrix $\sum \nolimits_{i}$. It is assumed that the random effects associated with the birds are independent of the errors $\varepsilon_{i}$. The Tukey-Cramer mean test was used to compare the means. All statistical analyses were performed using SAS statistical software (SAS 2010).

## Results and discussion

Table 1 shows that the highest T averages were detected in spring and summer, with 23.4 and 23 °C, respectively. The average T values of the external environment were 25.9 °C for spring, 25.5 °C for summer and 19.7 °C for autumn. It was observed that the T of the air-conditioned egg room were close to the external microclimatic conditions, and that only in autumn was the air-conditioned room within the ideal temperature range (10–20 °C) (Elibol and Brake, 2008a; Pokhrel et al. 2018). Therefore, it can be inferred that the air-conditioning system of the room was not efficient, thus, this T may increase productive losses during the storage of fertile eggs. It is important to emphasize that the seasons (spring and summer) were considered as critical points for embryo well-being. Thus, these were the hottest periods of the year and those that provided inadequate thermal conditions for the storage of fertile eggs in the hatchery.

**Table 1:** Microclimatic variables of the hatchery egg room in different seasons

Seasons of the year	Microclimatic variables		
	Temperature (T. °C)	Relative humidity (UR. %)	Specific enthalpy (h. kJ.kg of dry air^− 1^)
Spring	23.4 A	74.1 C	57.0 B
Summer	23.2 A	80.4 B	59.0 A
Autumn	20.0 B	83.4 A	50.6 C
Test F	97.11	64.54	71.30
p	< 0.0001	< 0.0001	< 0.0001
CV (%)	9.97	12.51	12.92
Standard deviation	2.22	9.90	7.20
Standard error	0.011	0.05	0.04

Means with different capital letters in the column differ from each other at a 5% probability level using the Tukey–Kramer test

During these times of the year, climate control must be carefully managed to prevent production losses due to high temperatures. Therefore, when fertile eggs are at high temperatures, embryonic development may begin in the egg room (Fiúza et al. 2006; Nazareno et al. 2015). It is very important that embryo development begins in the incubator to minimize losses in hatchability and chick quality.

The arrest of this embryonic development occurs when eggs are stored within the ideal T ranges, that is, below the physiological zero point (Tona et al. 2001; Pokhrel et al. 2018; Abioja et al. 2021). In addition, there are other egg losses which can be minimized during storage by using temperature control, such as: physical-chemical losses (albumen quality and pH), carbon dioxide, water loss and weight (Pleti et al. 2009; Nazareno et al. 2014; Pokhrel et al. 2018; Abioja et al. 2021).

When evaluating the quality of eggs stored under different times and environmental conditions, it was found that the greatest losses in relation to the internal quality of the eggs (haugh unit, yolk and albumen) were observed in environments where the temperature was above 20 °C (Barbosa et al. 2008a; Fernandes et al. 2015; Özlü et al. 2021; Zhao et al. 2024). Therefore, in order to maintain the quality of the eggs for a longer period, they must be stored in air-conditioned environments below 20 °C (Freitas et al., 2011; Elibol and Brake 2008a; Pokhrel et al. 2018).

Regarding RH, the highest average was observed in autumn with 83.4% and the lowest was detected in spring, with 74.1%, respectively. The spring period was the only one that was within the ideal RH ranges (55–75%) (Elibol and Brake 2008a; Pokhrel et al. 2018). In the external environment, the average RH values were 81.2% for spring, 85.7% for summer and 71.1% for autumn, respectively. It was noted that the external environment presented the highest averages in relation to the air-conditioned room. Therefore, during the air conditioning of the egg room, the air was dehumidified and the room cooling process began.

This phenomenon is common in air-conditioned systems (Luo et al. 2024). It is essential to control the RH inside the air-conditioned room during the storage of fertile eggs, as this will prevent condensation or evaporation of water from the eggs. In such case, when eggs are stored above the ideal range, water condensation will occur on the eggshells, which will favor conditions for the growth and proliferation of microorganisms (fungi and bacteria) (Gast and Holt, 2000; Lourens et al. 2006; Baracho et al. 2010). However, when eggs are stored below the ideal range, excessive water loss and a decrease in their weight and physical-chemical traits will occur (Elibol and Brake 2008a; Pokhrel et al. 2018; Abou-Kassem et al. 2024). Furthermore, inadequate storage of fertile eggs makes incubation unfeasible, thus increasing the number of contaminated eggs, malformations and embryonic mortality, and also reducing the production and quality of chicks.

Regarding specific enthalpy, it was found that the highest average occurred in summer with 59.0 kJ.kg of dry air-1 and the lowest average was detected in autumn, with 50.6 kJ.kg of dry air-1, respectively. Therefore, it was evident that all seasons of the year were above the ideal ranges (25 to 44.7 kJ.kg of dry air-1) according to Rodrigues et al. (2011). In the external environment, the average specific enthalpy values were 68.1 kJ.kg of dry air-1 for spring, 69.2 kJ.kg of dry air-1 for summer and 44.0 kJ.kg of dry air-1 for autumn, respectively. Based on these results, it is concluded that the amount of heat in the egg room was greater than recommended; these high specific enthalpy values were attributed to the rise in temperature alongside with the relative humidity. With adverse temperature and relative humidity conditions, egg storage can be affected. Koelkebeck et al. (2008); Patterson et al. (2008); Fernandes et al. (2015) observed that, during the summer, the quality and shelf life of eggs were reduced due to the adverse conditions to which these eggs were exposed; they also had an older appearance, which hinders egg hatching (Barbosa et al. 2008b).

Table 2 showed a difference (*p* < 0.0001) between the ages of broiler breeders and water loss of fertile eggs in the air-conditioned room. The highest average water loss of eggs was observed in eggs from old breeders, with 1.05%, which can be explained because eggs from old breeders lose more water, due to the fact that they are larger, have a thinner shell and greater porosity, compared to eggs from new breeders (Nazareno et al. 2015, 2014; Feng et al. 2020).

**Table 2:** Water losses from fertile eggs at different ages of breeders in the climate-controlled room of the hatchery

Breeders’ ages	Water loss (%)
Young	0.83 C
Intermediate	0.93 B
Old	1.05 A
Test F	428.17
p	< 0.0001
CV (%)	2.443
Standard deviation	10.103
Standard error	0.284

Means with different capital letters in the column differ from each other at a 5% probability level using the Tukey–Kramer test

Water losses from eggs are directly related to egg size and weight, which increase with the age of the breeders (Nazareno et al. 2014; Zhao et al. 2024; Maina et al. 2025). However, shell weight does not increase to the same extent (Agbehadzi et al. 2024; Zhao et al. 2024). Therefore, as the bird ages, shell thickness and its percentage in relation to egg weight decrease (Tanure et al. 2009; Corrêa et al. 2011; Feng et al. 2020). As a result, eggs from old breeders showed greater water loss and poorer quality (Feng et al. 2020; Zhao et al. 2024). In order to reduce these water losses while the eggs are in the air-conditioned room, it is important that the microclimatic conditions are effectively controlled, which did not happen in this study.

In Table 3, it was possible to verify a difference (*p* < 0.0001) between the different storage times for water loss of fertile eggs in the climate-controlled egg room of the hatchery. The longest storage times (244–255, 220–231 and 198–208 h) presented high water losses, with average values of 1.60, 1.41 and 1.23%, respectively. Therefore, the water loss of the eggs was influenced by the storage time of the eggs in the climate-controlled room of the hatchery, which corroborates with the authors Reijrink et al. (2010), Pokhrel et al. (2018); Melo et al. (2021), who observed that the longer the storage time of fertile eggs, the greater the water loss of the eggs. This does not mean that eggs should be incubated immediately after laying, it is known that a short storage period prevents embryonic mortality (Reijrink et al. 2010; Nasri et al. 2020; Dai et al. 2025).

**Table 3:** Water loss from fertile eggs during different storage times in the hatchery’s air-conditioned room

Storage times (h)	Water loss (%)
87–103	0.69 D
174–184	1.11 C
196–208	1.26 B
244–255	1.60 A
63–77	0.56 D
150–160	0.90 C
172–184	1.12 B
220–231	1.41 A
40–57	0.39 D
128–137	0.79 C
150–161	0.89 B
198–208	1.23 A
Test F	1323.15
p	< 0.0001
CV (%)	2.443
Standard deviation	10.103
Standard error	0.284

Means with different capital letters in the column differ from each other at a 5% probability level using the Tukey–Kramer test

Studies have reported that storing fertile eggs for a period of three to four days in a climate-controlled environment improves hatchability (Decuypere et al. 2001; Nasri et al. 2020). However, with the increase in the storage time of commercial eggs, it was noted that some elements of the eggs (yolk, Haugh unit, viscosity and albumen pH) degraded, as well as water loss increased (Barbosa et al. 2008a; Fernandes et al., 2015; Zhao et al. 2024). Therefore, storage times alongside with the traits and water loss of the eggs can be influenced by the microclimatic conditions of the climate-controlled egg room, subsequently influencing the hatchability and quality of the chicks.

In short, eggs from older hens lose more water than those from intermediate and new hens. Therefore, the longer the eggs arethe incubator environment across seasons stored, the greater the water loss from the eggs. It is very important that companies pay attention to this context, since eggs from new hens are more suitable for longer storage periods, compared to eggs from old hens. This is because the latter are more resistant to water loss, in addition to having better albumen quality. Excessive water loss during long storage periods has been related to the reduction and loss of egg hatchability (Elibol and Brake 2008b; Moraes et al. 2009). Therefore, by reducing storage time, the hatchery will be able to avoid excessive water loss, which will consequently increase the hatching rate and reduce the occurrence of poor quality chicks (attributed to inadequate water loss, poor healing of the navels, sticky down, and increased hatch window).

The microclimatic variables (T, RH and h) of the incubator differed significantly (*p* < 0.0001, *p* = 0.0002 and *p* = 0.0004, respectively; Table 4) in the different seasons of the year (spring, summer and autumn). The highest average temperature was detected in summer, with 37 °C. The average temperature of the incubator room was 25.2 °C for spring, 26.9 °C for summer and 20.5 °C for autumn. In general, the variations between the incubator temperatures were: 11.6; 10.1 and 16.2 °C for spring, summer and autumn.

**Table 4:** Microclimatic variables of the incubator environment across seasons

Seasons of the year	Air temperature (T. °C)	Relative humidity (UR. %)	Specific enthalpy (h. kJ.kg of dry air-1)
Spring	36.8 B	51.6 B	87.8 B
Summer	37.0 A	55.0 A	90.5 A
Autumn	36.7 B	54.5 A	90.0 A
Test F	187.39	69.11	54.04
p	< 0.0001	0.0002	0.0004
CV (%)	1.53	14.85	8.52
Standard deviation	0.564	7.973	7.624
Standard error	0.003	0.044	0.042

Means with different capital letters in the column differ from each other at a 5% probability level using the Tukey–Kramer test

It was found that the temperature conditions in the incubator room were lower, as they represent the standard room temperature. Therefore, although the incubator is a controlled environment, it was observed that it was unable to maintain the internal temperature within the ideal ranges (37.5 to 37.8 °C) (Elibol and Brake, 2008a; Tanure et al. 2009; Abioja et al. 2021). Temperature directly impacts embryo development, egg hatching and, subsequently, broiler chick development. Temperature variations in the incubator may lead to an increase in the mortality rate and embryonic malformations, in addition to increasing the chick hatch window. Therefore, when the incubator temperature is below the ideal range, there will be variations in the duration of egg incubation, because the metabolic rate of the embryo is directly related to the incubation temperature; therefore, there will be a delay in development and an increase in mortality in the first days (Van Brecht et al. 2005; Abioja et al. 2021; Özlü et al. 2021). However, when there are high variations (up to 1.5 °C) from the ideal ranges, this can cause variations in the temperature of the microenvironment around the egg that will compromise hatching, accelerate embryonic development, compromise the correct position of the embryos and increase their mortality in the last days of incubation (Van Brecht et al. 2005; Abioja et al. 2021; Özlü et al. 2021).

The highest average relative humidity was detected in the summer, at 55%. The average relative humidity of the incubator room environment was 79% in the spring, 84.1% in the summer and 63.1% in the fall. The relative humidity conditions in the incubator room were higher than those inside the incubator. Thus, the incubators were able to maintain relative humidity within the ideal range of 50 to 65%, as recommended in the literature (Elibol and Brake, 2008a; Tanure et al., 2009) during the seasons studied. It can therefore be inferred that the incubators were efficient in maintaining relative humidity within the recommended range. During incubation, the egg loses 11 to 13% of water, which is why controlling relative humidity is so important, as excessive water loss can delay the hatching of embryos (Tanure et al., 2009; Abioja et al. 2021; Özlü et al. 2021). For better incubation performance, relative humidity must be within the ideal range. Therefore, when the relative humidity of the incubator is below the ideal range, excessive moisture loss will occur from the embryos, impairing hatching and resulting in small, dehydrated chicks. However, if the relative humidity is above the ideal range, the embryos tend to hatch early and are often wet, and residual albumen may also occur and may cause late embryonic mortality (Tanure et al., 2009; Abioja et al. 2021; Özlü et al. 2021).

The highest average specific enthalpy was observed in summer when compared to other seasons of the year. The specific enthalpies in the incubator room were lower in relation to the internal environment of the incubator. It was also observed that the specific enthalpy of the incubators was outside the ideal range (92.6 to 110.6 kJ.kg of dry air-1) in relation to what was proposed in this study, according to the ideal conditions of temperature and relative humidity, during the seasons of the year studied. This deviation from the ideal ranges was caused by the low temperature values in the incubator. Summer was the season that was closest to the thermal comfort conditions for the incubation of fertile eggs in relation to the other seasons studied. It is also important to emphasize that the autumn period can be considered critical for the embryo, since the incubators are not able to convert the external temperature of the incubation room to the ideal conditions, providing the worst thermal conditions for incubation of fertile eggs. Therefore, it was found that the incubator was working inefficiently, as the temperature and specific enthalpy values were outside the ideal conditions, which could increase production losses during this stage.

In Table 5, a difference (*p* < 0.0001, *p* = 0.0083 and *p* = 0.0209, respectively) was observed between the storage times for temperature, relative humidity and specific enthalpy. The highest average temperature was detected in storage times A3 (196–208, 172–184, 150–161 h), with 37.1 °C and A1 (87–103, 63–77, 40–57 h), with 37.0 °C. It was observed that the temperature for the different storage times inside the incubator was below the ideal ranges (37.5 to 37.8 °C), according to Elibol and Brake (2008a), Tanure et al. (2009), Abioja et al. (2021) and Özlü et al. (2021). This can compromise the incubation of the eggs, delaying hatching and increasing the hatching window of chicks, which can cause losses in hatchability and quality of the broiler chicks. Storage times A3 and A1 were those that had the best temperature conditions during incubation, in relation to the other storage times.

**Table 5:** Incubator microclimatic variables for different storage times

Seasons of the year	Microclimatic variables		
	Temperature (T. °C)	Relative humidity (UR. %)	Specific enthalpy (h. kJ.kg of dry air^− 1^)
A1	37.0 A	53.6 B	90.2 A
A2	36.5 C	55.2 A	89.7 A
A3	37.1 A	52.0 C	88.7 B
A4	36.7 B	54.0 B	89.1 B
Test F	403.03	13.13	8.49
p	< 0.0001	0.0083	0.0209
CV (%)	1.53	14.85	8.52
Standard deviation	0.564	7.973	7.624
Standard error	0.003	0.044	0.042

A1 = 87–103, 63–77, 40–57 h; A2 = 174–184, 150–160, 128–137 h; A3 = 196–208, 172–184, 150–161 h; A4 = 244–255, 220–231, 198–208 h

Means with different capital letters in the column differ from each other at a 5% probability level using the Tukey–Kramer test

The highest average relative humidity was verified in storage times A2 (174–184, 150–160, 128–137 h), with 55.2%. It was noted that all relative humidities of the storage times were within the ideal ranges (50 to 65%), according to Elibol and Brake (2008a), Tanure et al. (2009), Abioja et al. (2021) and Özlü et al. (2021). This demonstrated that relative humidity was not a factor that may have compromised the incubation of these eggs.

The highest average specific enthalpy was observed in storage times A1 (87–103, 63–77, 40–57 h), with 90.2 kJ.kg of dry air-1. It was noted that the specific enthalpies in all storage times were below the ideal ranges (92.6 to 110.6 kJ.kg of dry air-1). These values below the ideal ranges were caused by the low average temperature value in the incubator. Therefore, storage time A1 was the one that was closest to the thermal comfort conditions for the incubation of fertile eggs in relation to the others.

Table 6 shows the difference (*p* < 0.0001) between all breeder ages in the water loss of fertile eggs in the incubator environment. The highest average water loss from eggs was detected in the old breeder age with 12.35%. The greater water loss during incubation for eggs produced by old breeders can be justified by the greater porosity and thinner shell thickness (Agbehadzi et al. 2024; Zhao et al. 2024). The reduction in shell quality, combined with the increase in the age of the breeder, results in greater egg weight loss during incubation, in addition to increasing the embryonic mortality rate. As a consequence, a decrease in egg hatchability is observed (Tona et al. 2001; Pokhrel et al. 2018; Abioja et al. 2021). Shell quality is a crucial factor for good incubation performance. The main reason for the reduced hatchability of eggs from older breeders is the deterioration in shell quality.

**Table 6:** Water loss from fertile eggs at different ages of breeders in the incubator environment

Breeders’ ages	Water loss (%)
Young	9.45 C
Intermediate	11.81 B
Old	12.35 A
Test F	1219.82
p	< 0.0001
CV (%)	16.28
Standard deviation	1.830
Standard error	0.051

Means with different capital letters in the column differ from each other at a 5% probability level using the Tukey–Kramer test

The main reason for the reduction in hatchability of eggs from older breeders is due to reduced shell quality. When incubating eggs from older breeders, it is necessary to consider a higher incubation humidity in order to avoid excessive dehydration of the eggs (Costa et al. 2011; Agbehadzi et al. 2024; Zhao et al. 2024).

It was noted that water losses of fertile eggs from older and intermediate breeders were within the ideal ranges (12 and 13% water loss) from the time of incubation until transfer for hatching, with losses between 11% and 14% also being acceptable (Maudin, 1993). However, Hays and Spear (1951) observed that satisfactory hatchability results occurred when the weight loss of incubated eggs, assessed at 17 days, did not exceed 12%. On the other hand, Rosa et al. (2002) concluded that water loss between 11% and 12%, up to 18 days of incubation, provided better hatchability. Ferreira et al. (2006), Tanure et al. (2009) and Grochowska et al. (2019) obtained similar results, observing that the greatest water losses during incubation occurred in eggs from older breeders.

In Table 7, a difference (*p* < 0.0001) was observed between the different storage times for water loss of fertile eggs during incubation. The longest nested storage times (244–255, 220–231 and 198–208 h) were those that presented the greatest water losses, with average values of 13.35, 12.45 and 11.61%. The greatest water losses of eggs during incubation occurred with the increase in the storage period, and these results are in agreement with those found by Grochowska et al. (2019).

**Table 7:** Water loss from fertile eggs during different storage times in the incubator environment

Storage times (h)	Water loss (%)
87–103	10.43 C
174–184	11.61 B
196–208	11.81 B
244–255	13.35 A
63–77	9.88 C
150–160	11.10 B
172–184	11.10 B
220–231	12.45 A
40–57	9.27 C
128–137	10.62 B
150–161	10.81 B
198–208	11.61 A
Test F	139.97
p	< 0.0001
CV (%)	16.28
Standard deviation	1.830
Standard error	0.051

Means with different capital letters in the column differ from each other at a 5% probability level using the Tukey–Kramer test

Similarly, a study carried out with heavy Cobb breeders (33 and 58 weeks of age) concluded that the greatest weight loss occurred in eggs produced by older birds after eight days of storage (Ferreira et al. 2006). Furthermore, Tanure et al. (2009) observed that the greatest water losses from fertile eggs from light breeders during incubation occurred during the longest storage times. It is worth emphasizing that stored eggs may have a longer incubation period and delayed embryonic development (Reis et al. 1997; Nasri et al. 2020; Dai et al. 2025).

In summary, it was noted that eggs from older hens presented greater water loss compared to those from intermediate and new hens. Furthermore, it was found that the longer the storage time of the eggs, the greater the water loss observed.

### Limitations and implications

This study was conducted under commercial hatchery conditions, which enhances the practical relevance of the findings but limits the control of certain experimental variables. Specifically, storage time intervals were defined according to operational routines rather than standardized experimental design, and microclimatic conditions could not be fully homogenized across all treatments. Additionally, hatchability was not directly measured, which restricts the ability to establish causal relationships between the observed effects and final incubation performance.

Despite these limitations, egg water loss represents a key physiological and biophysical indicator of embryonic development, being directly associated with eggshell conductance, water vapor diffusion, and gas exchange processes during storage and incubation. The results demonstrate that temperature, relative humidity, and specific enthalpy act in an integrated manner to influence moisture gradients between the egg and the surrounding environment, while breeder age modulates these responses through structural changes in eggshell porosity and thickness. Thus, the patterns observed are supported by well-established mechanistic relationships.

From an applied perspective, the findings highlight the need for improved environmental control in egg storage rooms, particularly under tropical conditions, where deviations from recommended microclimatic ranges were consistently observed. Adjustments in storage duration according to breeder age, along with tighter control of temperature and humidity, may help reduce excessive water loss and improve incubation efficiency. Future studies should integrate direct hatchability measurements and controlled experimental designs to further elucidate the causal pathways linking microclimate, egg physiology, and production outcomes.

## Conclusion

The microclimatic conditions in the egg room remained above recommended ranges across all seasons under tropical conditions, with the most critical deviations occurring during summer, while incubator conditions were generally below recommended values. Egg water loss was higher in eggs from older breeders and under prolonged storage times, whereas lower losses were associated with shorter storage durations, particularly in eggs from younger breeders.

From a practical standpoint, improving environmental control in egg storage rooms, reducing storage duration, and adopting age-specific management strategies, such as prioritizing faster incubation of eggs from older flocks and adjusting incubator humidity, are essential to minimize excessive water loss. Although hatchability was not directly measured, these results indicate that optimizing microclimate and storage conditions may contribute to improved incubation efficiency and chick quality.

## Data Availability

The data supporting the findings of this study are available from the corresponding author upon reasonable request.

## Declarations

During the preparation of this work, the authors utilized ChatGPT to improve readability and language of the manuscript. After using this tool, the authors carefully reviewed and edited the content as needed and take full responsibility for the publication’s content.

## References

[CR1] Abioja MO, Abiona JA, Akinjute OF, Ojoawo HT (2021) Effect of storage duration on egg quality, embryo mortality and hatchability in FUNAAB-ɑ chickens. J Anim Physiol Anim Nutr (Berl) 105:715–724 10.1111/jpn.13480 33377577

[CR2] Abou-Kassem DE, El-Sayiad RA, El-Hack MEA, Kamal TAHAAE, Alfassam M, Rudayni HE, Allam HA, Moustafa AA, Algopopishi M (2024) Ashour, E. Impacts of storage period and egg weight on hatching and growth performance of growing Japanese quails. Poult Sci 103:103772 10.1016/j.psj.2024.103772 PMC11063647 38669822

[CR3] Agbehadzi RK, Meteyake HT, Adjei-Mensah B, Sasu P, Koranteng AA, Dassidi N, Hamidu JA, Tona K (2024) Effects of layer breeder age and reduced incubator oxygen concentrations on embryo development, hatching events, chick quality, embryonic mortality and hatchability of fertile eggs. Heliyon 10:e40468 10.1016/j.heliyon.2024.e40468 PMC11625139 39650182

[CR4] Almeida JG, Vieira SL, Gallo BB, Conde ORA, Olmos AR (2006) Period of incubation and posthatching holding time influence on broiler performance. Revista Brasileira de Ciência Avícola, Campinas, v. 8, n. 3, pp. 153–158, a

[CR5] Baracho MS, Nääs IA, Gigli ACS (2010) Impacto das variáveis ambientais em incubatório de estágio múltiplo de frangos de corte. Engenharia Agrícola Sorocaba 30:563–577

[CR6] Barbosa NAA, Sakomura NK, MEndonça MO, Freitas ER, Fernandes JB (2008a) K. Qualidade de ovos comerciais provenientes de poedeiras comerciais armazenados sob diferentes tempos e condições de ambientes. Porto Alegre, vol 24. Archives of Veterinary Science, pp 127–133. 2

[CR7] Barbosa VM, Cançado SV, Baião NC, Lana AMQ, Lara LJC, Souza MR (2008b) Efeitos da umidade relativa do ar na incubadora e da idade da matriz leve sobre o rendimento da incubação. Arquivo Brasileiro de Medicina Veterinária e Zootecnia, Belo Horizonte, v.60, n.3, pp. 741–748

[CR8] Brake JT (1996) Optimization of egg handling and storage, vol 12. World Poultry Science, Amsterdam, pp 6–9

[CR9] Corrêa AB, Silva MA, Corrêa GSS, Santos GG, Felipe VPS, Wenceslau RR, Souza GH, Campos NC (2011) F.L. Efeito da interação idade da matriz x peso do ovo sobre o desempenho de codornas de corte. Arquivo Brasileiro de Medicina Veterinária e Zootecnia, Belo Horizonte, v.63, n.2, pp. 433–440

[CR10] Costa CHR, Barreto SLT, Gomes PC, Hosoda IH, Lipari CA, Lima HJA (2011) Níveis de fósforo disponível em dietas para codornas japonesas de 45 a 57 semanas de idade, vol 40. Revista Brasileira de Zootecnia, Viçosa, pp 2152–2160. 10

[CR11] Dai D, Wang X, Wu K, Lan F, Jin J, Zhang W, Wen C, Li J, Yahg N, Sun C (2025) Proteomic and N-glycosylation analysis of fertile egg White during storage and incubation in chickens. Poult Sci 104:104526 10.1016/j.psj.2024.104526 PMC11635699 39608286

[CR12] Decuypere E, Tona K, Bruggeman V, Bamelis E (2001) The day-old chick: a crucial hinge between breeders and broilers, vol 57. World’s Poultry Science Journal, Cambridge, pp 127–138

[CR13] Elibol O, Brake J (2008a) Effect of egg weight and position relative to incubator fan on broiler hatchability and chick quality, vol 87. Poultry Science, Savoy,, pp 1913–1918 10.3382/ps.2008-00014 18753462

[CR14] Elibol O, Brake J (2008b) Effect of egg position during three and fourteen days of storage and turning frequency during subsequent incubation on hatchability of broiler hatching eggs. Poultry science, Savoy, v. 87, n. 6, p. 1237–1241 10.3382/ps.2007-00469 18493016

[CR15] Fasenko GM (2007) Egg storage and the embryo. Poult Sci Champaign v 86:1020–1024 10.1093/ps/86.5.1020 17435042

[CR16] Feng J, Zhang HJ, Wu SG, Qi GH, Wang J (2020) Uterine transcriptome analysis reveals mRNA expression changes associ ated with the ultrastructure differences of eggshell in young and aged laying hens. BMC Genom 21:770 10.1186/s12864-020-07177-7 PMC7654033 33167850

[CR17] Fernandes DPB, Mori C, Nazareno AC, Pizzolante CC, Moraes JE (2015). Qualidade interna de diferentes tipos de ovos comercializados durante o inverno e o verão. Arquivo Brasileiro de Medicina Veterinária e Zootecnia (Online), v. 67, pp. 1159–1165

[CR18] Ferreira FC, Lara LJC, Baião NC, Lana AMQ, López CAA, Moraes DT (2006) Influência da idade da matriz e do período de armazenamento dos ovos sobre a uniformidade dos pintos aos 11 dias de idade. Revista Brasileira de Ciência Avícola, Campinas, supl. 8, p. 17

[CR19] Fiúza MA, Lara LJC, Aguilar CAL, Ribeiro BRC, Baião NC (2006) Efeitos das condições ambientais no período entre a postura e o armazenamento de ovos de matrizes pesadas sobre o rendimento de incubação. Arquivo Brasileiro de Medicina Veterinária e Zootecnia, Belo Horizonte, v.58, n.3, pp. 408–413

[CR20] Freitas LW, Paz I, Garcia RG, Caldara FR, Seno LO, Felix GA, Lima NDS, Ferreira VMOS, Cavichiolo F (2011) Aspectos qualitativos de ovos comerciais submetidos a diferentes condições de armazenamento. Revista Agrarian, pp 66–72. 4

[CR21] Gast RK and HOLT PS (2000) Influence of the level and location of contamination on the multiplication of *Salmonella enteritidis* at different storage temperatures in experimentally inoculated eggs, vol 79. Poultry Science, Savoy, pp 559–563 10.1093/ps/79.4.559 10780654

[CR22] Grochowska E, Kinal A, Sobek Z, Siatkowski I, Bednarczyk M (2019) Field study on the factors affecting egg weight loss, early embryonic mortality, hatchability, and chick mortality with the use of classification tree technique. Poult Sci 98:3626–3636 10.3382/ps/pez180 30993348

[CR23] Hays FA, Spear EW (1951) Losses in egg weight during incubation associated with hatchability, vol 30. Poultry Science, Savoy, pp 106–107

[CR24] Koelkebeck KW, Patterson PH, Anderson KE, Darre MJ, Carey JB, Ahn DU, Ernst RA, Kuney DR, Jones D (2008) Temperature sequence of eggs from oviposition through distribution: Processing—Part 2, vol 87. Poultry Science, Champaign, pp 1187–1194 10.3382/ps.2007-00239 18493010

[CR25] Liu H, Ma WWS, Ding J, Qu S, Ge Q, Wang MY (2024) Hierarchical triply periodic minimal surface shell lattices with superior isotropic elasticity: Design guidelines, fabrication, and validation. Additive Manuf 94:104451

[CR26] Lourens S, R. Van Den Brand Molenaar, Heetkamp H, Meijerhof MJWR, KEMP, B (2006) Effect of Egg Size on Heat Production and the Transition of Energy from Egg to Hatchling, vol 85. Poultry Science, Champaign, pp 770–776 10.1093/ps/85.4.770 16615362

[CR27] Luo J, Shen Y, Yang H (2024) Investigations on an integrated air-conditioning system using technologies of desiccant dehumidification, indirect evaporative cooling and CO2 capture. Appl Energy 369:123601. 10.1016/j.apenergy.2024.123601

[CR28] Maina AN, Schulze H, Kiarie EG (2025) Effects of lifetime feeding of hydrolyzed yeast to broiler breeders on egg production, quality, and hatchling atributes. Poult Sci 104:104826 10.1016/j.psj.2025.104826 PMC11795560 39848209

[CR29] Maudin JM (1993) Measuring incubation moisture weight loss. Int Hatch Pract Driffield v 8(1):47

[CR31] Melo EF, Araújo ICS, Triginelli MV, Castro FLS, Baião NS, Lara LJ (2021) C. Effect of egg storage duration and egg turning during storage on egg quality and hatching of broiler hatching eggs. Animal 15:100111 10.1016/j.animal.2020.100111 33573937

[CR32] Moraes TGV, Romão JM, Cardoso WM (2009) Parâmetros da incubação e componentes dos ovos de codornas japonesas para corte (*Coturnix japonica*) submetidos à estocagem em baixas temperaturas (7,5 ± 1°C). Semina: Ciências Agrárias, Londrina, v.30, n.1, p. 233–242

[CR33] Nasri H, van den Brand H, Najjar T, Bouzouaia M (2020) Egg storage and breeder age impact on egg quality and embryo development. J Anim Physiol Anim Nutr (Berl) 104:257–268 10.1111/jpn.13240 31691404

[CR34] Nazareno AC, Silva IJO, Vieira AMC, Vieira FMC (2014) Microclima, idade das matrizes e tempo de estocagem influenciando nas respostas produtivas de ovos férteis. Revista Brasileira de Engenharia Agrícola e Ambiental v 18:1172–1178

[CR35] Nazareno AC, Silva IJO, Vieira AMC, Vieira FMC (2015) Microclima, idades das matrizes e tempos de estocagem influenciam na perda de água dos ovos no transporte? Eng Agr-Jaboticabal 35:635–645

[CR36] Özlü S, Uçar A, Erkuş T, Nicholson AD, Elibol O (2021) Effects of turning and short period of incubation during long-term egg storage on embryonic development and hatchability of eggs from young and old broiler grandparent flocks. Poult Sci 100:101026 10.1016/j.psj.2021.101026 PMC7921000 33652245

[CR37] Patterson PH, Koelkebeck KW, Anderson KE, Darre MJ, Carey JB, Ahn DU, Ernst RA, Kuney DR, Jones DR (2008) Temperature sequence of eggs from oviposition through distribution: Production—Part 1, vol 87. Poultry Science, Champaign, pp 1182–1186 10.3382/ps.2007-00242 18493009

[CR38] Pereira AR, Angelocci LR, Sentelhas PC (2002) Agrometeorologia: fundamentos e aplicações práticas. Agropecuária, Guaíba, p 478

[CR39] Pleti AK, Lima JJ, Candido LMB (2009) Qualidade interna do ovo de avestruz após estocagem em temperatura ambiente e refrigerada. Ciência Rural, Santa Maria, v.39, n.6

[CR40] Pokhrel N, Cohen EB, Genin O, Ruzal M, Sela-Donenfeld D, Cinnamon Y (2018) Effects of storage conditions on hatchability, embryonic survival and cytoarchitectural properties in broiler from young and old flocks. Poult Sci 97:1429–1440 10.3382/ps/pex393 29452390

[CR41] Reijrink, IAM, Berghmans D, Meijerhof R, Kemp B, Van Den Brand H (2010) Influence of egg storage time and preincubation warming profile on embryonic development, hatchability, and chick quality, vol 89. Poultry Science, Savoy, pp 1225–1238 10.3382/ps.2009-00182 20460670

[CR42] Reis LH, Gama LT, Chaveiro Soares M (1997) Effects of short storage conditions and broiler breeder age on hatchability, hatching time, and chick weights. Poultry Sci Savoy 76:1459–1466 10.1093/ps/76.11.1459 9355138

[CR43] Rodrigues VC, Silva IJO, Vieira FMC, Nascimento ST (2011) A correct enthalpy relationship as thermal comfort index for livestock. Int J Biometeorol 55(3):455–459. Springer Berlin, Heidelberg 10.1007/s00484-010-0344-y 20607305

[CR44] Rosa PS, Guidoni AL, Lima IL, Bersch FXR (2002) Influência da temperatura de incubação em ovos de matrizes de corte com diferentes idades e classificados por peso sobre os resultados de incubação. Revista Brasileira de Zootecnia, Viçosa, v. 31, n. 2, pp. 1011–1016, Suplemento

[CR45] Santos JRGDL, Fornari CM, Téo MA (2007) Influência da qualidade da casca do ovo sobre índices de produtividade de um incubatório industrial. Ciência Rural, Santa Maria, v.37, n.2, p.524–527

[CR46] Sas Institute (2010) Statistical analysis system: Realease 9.2, (software). Cary, 620 p

[CR56] Silveira RMF, Junior SLC, Dilelis F, Nazareno AC, Ticiano JC, da Silva IJO (2026) Linking egg size, embryonic development, and post-hatch thermoregulatory responses in broilers from young and old breeders. Poult Sci 105(3):106403. 10.1016/j.psj.2026.106403 PMC12861143 41579596

[CR47] Souza AV, Leão APA, Silva CFG, Peixoto JV, Chaves BR, Andrade SC, Fassani EJ, Alvarenga RR, Zangeronimo MG (2025) Reproductive characteristics of Japanese quail hatched from eggs incubated at different temperatures. Theriogenology p. 236:1–7 10.1016/j.theriogenology.2025.01.022 39892068

[CR48] Tanure CBGS, Café MB, Leandro NSM, Baião NC, Stringhini JH, Gome NA (2009) Efeitos da idade da matriz leve e do período de armazenamento de ovos incubáveis no rendimento de incubação. Arquivo Brasileiro, vol 61. de Medicina Veterinária e Zootecnia, Belo Horizonte, pp 1391–1396. 6

[CR49] Tona K, Bamelis F, Coucke W, Bruggeman V, Decuypere E (2001) Relantionship between broiler breeder’s age and egg weight loss and embryonic mortality during incubation in large-scale conditions. Journal of Applied Poultry Research, Athens, v. 10, pp. 221–227

[CR50] Tona K, Bamelis F, De Ketelaere B, Bruggeman V, Moraes VM, Buyse J, Onagbesan O, Decuypere E (2003) Effects of egg storage time on spread of hatch, chick quality, and chick juvenile growth. Poult Sci 82(5):736–741. 10.1093/ps/82.5.736 12762394

[CR52] Van Brecht A, Hens H, Lemaire JL, Aerts JM, Degraeve P, Berckmans D (2005) Quantification of the heat exchange of chicken eggs. Poult Sci Association Bélgica v 84:353–361 10.1093/ps/84.3.353 15782902

[CR53] Verbeke G, Molenberghs G (2000) Linear mixed models for longitudinal data. Springer Science + Bussiness Media, New York

[CR54] Vieira AMC (2008) Modelagem simultânea de média e dispersão e aplicações na pesquisa agronômica. 176 p. Tese (Doutor em Estatística e Experimentação Agronômica) – Escola Superior de Agricultura Luiz de Queiroz, Universidade de São Paulo, Piracicaba, 2008

[CR55] Zhao DR, Gao LB, Gong F, Feng J, Zhang HJ, Wu SG, Wang J, Min Y (2024) N. TMT-based quantitative proteomic analysis reveals eggshell matrix protein changes correlated with eggshell quality in Jing Tint 6 laying hens of different age. Poult Sci v 103:103463 10.1016/j.psj.2024.103463 PMC10840124 38281332

